# TLR4 Activates the β-catenin Pathway to Cause Intestinal Neoplasia

**DOI:** 10.1371/journal.pone.0063298

**Published:** 2013-05-14

**Authors:** Rebeca Santaolalla, Daniel A. Sussman, Jose R. Ruiz, Julie M. Davies, Cristhine Pastorini, Cecilia L. España, John Sotolongo, Oname Burlingame, Pablo A. Bejarano, Sakhi Philip, Mansoor M. Ahmed, Jeffrey Ko, Ramanarao Dirisina, Terrence A. Barrett, Limin Shang, Sergio A. Lira, Masayuki Fukata, Maria T. Abreu

**Affiliations:** 1 Division of Gastroenterology, Department of Medicine, University of Miami, Leonard Miller School of Medicine, Miami, Florida, United States of America; 2 Department of Pathology, University of Miami, Leonard Miller School of Medicine, Miami, Florida, United States of America; 3 Department of Radiation Oncology, Sylvester Comprehensive Cancer Center, University of Miami, Leonard Miller School of Medicine, Miami, Florida, United States of America; 4 Division of Gastroenterology, Department of Medicine, Northwestern University, Feinberg School of Medicine, Chicago, Illinois, United States of America; 5 Immunology Institute, Mount Sinai School of Medicine, New York, New York, United States of America; Baylor University Medical Center, United States of America

## Abstract

Colonic bacteria have been implicated in the development of colon cancer. We have previously demonstrated that toll-like receptor 4 (TLR4), the receptor for bacterial lipopolysaccharide (LPS), is over-expressed in humans with colitis-associated cancer. Genetic epidemiologic data support a role for TLR4 in sporadic colorectal cancer (CRC) as well, with over-expression favoring more aggressive disease. The goal of our study was to determine whether TLR4 played a role as a tumor promoter in sporadic colon cancer. Using immunofluorescence directed to TLR4, we found that a third of sporadic human colorectal cancers over-express this marker. To mechanistically investigate this observation, we used a mouse model that over-expresses TLR4 in the intestinal epithelium (villin-TLR4 mice). We found that these transgenic mice had increased epithelial proliferation as measured by BrdU labeling, longer colonic crypts and an expansion of Lgr5+ crypt cells at baseline. In addition, villin-TLR4 mice developed spontaneous duodenal dysplasia with age, a feature that is not seen in any wild-type (WT) mice. To model human sporadic CRC, we administered the genotoxic agent azoxymethane (AOM) to villin-TLR4 and WT mice. We found that villin-TLR4 mice showed an increased number of colonic tumors compared to WT mice as well as increased β-catenin activation in non-dysplastic areas. Biochemical studies in colonic epithelial cell lines revealed that TLR4 activates β-catenin in a PI3K-dependent manner, increasing phosphorylation of β-catenin^Ser552^, a phenomenon associated with activation of the canonical Wnt pathway. Our results suggest that TLR4 can trigger a neoplastic program through activation of the Wnt/β-catenin pathway. Our studies highlight a previously unexplored link between innate immune signaling and activation of oncogenic pathways, which may be targeted to prevent or treat CRC.

## Introduction

Colorectal cancer (CRC) is the second and third most common type of cancer in women and men, respectively, and accounts for more than half of the cancer diagnoses in developed countries [1]. Implementation of screening strategies has reduced death from CRC but over a million people in the US continue to be affected yearly [2], [3]. Although the genetic underpinnings of CRC have been extensively studied, this has not yet resulted in novel and safe preventive strategies for average risk patients, without a family history of CRC or clinical risk factors [4], [5]. Colonic bacteria and low-grade inflammation have been implicated in the development of sporadic CRC and could serve to explain why cancer of the colon is significantly more common than cancer of the small intestine where the bacterial density is lower [6]. Given that the innate immune system is the interface between the host and the microbiota, we set out to understand whether innate immune signaling could engender colon cancer with the goal of identifying new avenues for prevention or treatment of CRC.

We have previously identified a link between toll-like receptor signaling in inflammatory bowel disease (IBD) and colitis-associated cancer (CAC) [7]. Our studies showed that the majority of dysplasia and cancers occurring in the setting of IBD demonstrate over-expression of toll-like receptor 4 (TLR4)–the receptor for lipopolysaccharide (LPS) from Gram-negative bacteria [7]. The apical surface of the intestinal epithelium is exposed to LPS from the lumen. There is a certain rate of bacterial translocation and exposure to LPS on the basolateral side of the epithelium, especially during epithelial injury, which may turn on inflammatory responses through TLR4 signaling [8]. In mouse models, we showed that TLR4 knock-out mice are protected against CAC [9], whereas mice over-expressing TLR4 in the intestinal epithelium are prone to developing inflammatory neoplasia [7].

In humans, both genetic epidemiologic data and immunohistochemical associations support a role for TLR4 in CRC. First, a polymorphism increasing TLR4 signaling results in more aggressive CRC [10] whereas polymorphisms that decrease TLR4 signaling are protective against CRC [11]. Second, high expression of TLR4 correlates with more advanced grades of colonic neoplasia [7], [12], [13]. Furthermore, TLR4 expression is associated with lower overall survival and the presence of liver metastases in humans, and CRC relapse following treatment is predicted by high levels of stromal TLR4, lending credence to the further mechanistic study of this marker [12], [13]. Finally, silencing of TLR4 with RNA interference in xenograft models of CRC decreases the metastatic tumor burden in the liver, supporting a therapeutic potential in targeting TLR4 [14]. These observations compelled us to understand mechanistically how TLR4 contributes to the pathogenesis of CRC.

Most colonic tumors carry mutations in Wnt pathway genes, such as the adenomatous polyposis coli (APC) gene, that result in β-catenin activation [15]. Despite activating Wnt pathway mutations, most colon cancers demonstrate only variable expression of nuclear β-catenin–a hallmark of Wnt pathway activation and cancer stem cells [16], [17]. These observations suggest that even in the setting of APC mutations, microenvironmental factors may be required to stabilize β-catenin and promote tumor growth [18], [19]. Moreover, studies have shown that in addition to genetic mutations leading to constitutive Wnt pathway activation, other signaling pathways can stabilize β-catenin [20].

In the current study, we query the role of TLR4 as a tumor promoter in the intestine. We show that a subset of adenomas–a precursor of colon cancer, and sporadic CRCs have increased epithelial expression of TLR4. We demonstrate experimentally that TLR4 can activate the central program of cell proliferation in the intestine, the β-catenin pathway, and that over-expression of TLR4 is sufficient to make the intestinal epithelium tumor prone. The implications from our study are far-reaching since our data suggest that bacterial signaling pathways synergize with oncogenic pathways to promote CRC.

## Methods

### Ethics Statement

The human tissue microarrays used for this manuscript are considered non-human samples for research. This tissue was obtained de-identified from US Biomax, Inc. (www.biomax.us). The authors were not in contact with any of the human subjects, nor did they receive any information from those subjects. The only available information about the samples was sex, age, type of tissue and pathology diagnosis. The Human Subject Research Office at the University of Miami approved our project as non-human subject research, agreeing that our study did not require IRB approval.

All experiments using mice were performed according to the University of Miami Institutional Animal Care and Use Committees’ (IACUC) guidelines. Mouse colonoscopies were performed under anesthesia, and all efforts were made to minimize suffering.

### Mouse Models

C57BL/6J and villin-TLR4 mice were bred in specific-pathogen free (SPF) conditions and fed under free access to a standard diet and water. The villin-TLR4 transgenic mice were generated as previously described [21] and express a TLR4 transgene in the intestinal epithelium. Lgr5-EGFP mice (B6.129P2-Lgr5^tm1(cre/ESR1)Cle^/J) were purchased from Jackson Laboratories (Bar Harbor, ME), and were crossed to our villin-TLR4 mice.

We used two models of intestinal tumorigenesis, AOM and AOM-DSS. AOM causes DNA alkylation leading to mutations, and DSS is used to induce colitis. Mice were used between 7 to 12 weeks of age. To induce tumorigenesis with AOM alone the mice received 6 injections of AOM (14.8mg/kg i.p.) once a week for 6 weeks (Figure S1). We performed the mouse colonoscopies right before they were sacrificed for tissue collection, after week 17. In the AOM-DSS model, mice were injected once with a dose of AOM (14.8mg/kg i.p. on day 0) followed by 3 cycles of 2% DSS as previously described [7].

### Histology and Immunohistochemistry

Both human and mouse tissue were fixed in buffered 10% formalin and embedded in paraffin. The tissue was cut 5 µm thick, and the slides were deparaffinized and rehydrated. Antigen retrieval was performed in heated 10mM citrate buffer. Histologic features were analyzed by hematoxylin and eosin staining. The formalin-fixed paraffin-embedded human tissue that we analyzed was purchased from US Biomax, Inc. (http://www.biomax.us/) as tissue microarrays. All the arrays contained colon tissue from sporadic adenocarcinomas, and normal surrounding tissue, adenomas, chronic inflammation of the mucosa and random normal tissue. Immunofluorescence was performed to detect TLR4 (anti-human TLR4, Novus Biologicals), cytokeratin as a marker for epithelial cells (anti-human pan-cytokeratin, Abcam), and DAPI to counterstain the nuclei. TLR4 detection was enhanced using the Tyramide-conjugated fluorochrome Alexa Fluor 488 (Invitrogen). Pan-cytokeratin was detected using an anti-rabbit secondary antibody conjugated with Alexa Fluor 647 (Invitrogen). Tissue microarrays were analyzed using a HistoRX PM-2000 reader, which is a microscope-based multiplex imaging device. Negative controls were performed omitting the primary antibodies (pan-cytokeratin and TLR4), to ensure proper staining and avoid evaluating background (Figure S2).

For the murine samples, immunohistochemistry was performed to detect total β-catenin (anti-mouse β-catenin, Sigma-Aldrich), β-catenin^Ser552^ [22], BrdU (anti-BrdU kit, Invitrogen) and cyclin-D1 (anti-mouse/human cyclin-D1, Dako); all stains used horseradish peroxidase conjugated antibody, with chromogenic detection with the substrate 3-3′-diaminobenzidine, and finally counterstained with hematoxylin.

Samples collected from Lgr5-EGFP mice were fixed overnight in buffered 4% paraformaldehyde. Next the tissue was cryopreserved incubating the samples in 30% sucrose for 6h and then embedded in optimal cutting temperature (OCT) medium and frozen in a liquid nitrogen/2-methylbutane bath. Immunofluorescence was performed to detect EGFP (anti-GFP Antibody, Abcam; Negative control without primary antibody: Figure S3b), the intestinal epithelium marker EpCAM (anti-mouse EpCAM, Biolegend); all counterstained with nuclear staining with DAPI. Images of the immunofluorescence staining were obtained using a Leica TCS SP5 Confocal Microscope.

Mouse immunostained tissue samples were counted and scored by different investigators masked to the mouse genotype and treatment. In addition, we scored the degree of histologic inflammation in the mouse tissue used in this study. The samples were scored as previously described [7], by three pathologists (O.B., P.B., N.R.) blinded to genotype and treatment. Human tissue microarrays were analyzed and scored by two investigators (R.S., D.S.) blinded to the histopathologic diagnosis of each sample. We developed a TLR4 scoring system that consisted in grading the intensity of TLR4 in IEC as negative (score 0), low positive (score 1), positive (score 2), and high positive (score 3).

### Cell Lines

The human adenocarcinoma cell line SW480, and non-transformed rat IEC cell line IEC-6 were obtained from ATCC (CCL-228™ and CRL-1592™). Cells were grown in DMEM 10% fetal bovine serum at 37°C in an incubator with controlled 5% CO_2_. To analyze protein expression the cells were seeded in 6-well plates, stimulated with LPS 1 µg/ml and the cell lysate was collected at different time points (30, 60, 90 and 120 minutes, and 4, 6 and 24 hours) as stated in the legends.

Nuclear protein was extracted by incubating cells with a protease inhibitor cocktail and lysis buffer (10****mM HEPES, 1.5****mM MgCl_2_, 10****mM KCl, 0.5 mM DTT, 0.05% Tergitol®, pH 7.9). After centrifugation at 3000****rpm for 10****min, the supernatant was discarded. The pellet, containing the nuclei, was homogenized in the nuclear lysis buffer (5****mM HEPES, 1.5****mM MgCl_2_, 0.2****mM EDTA, 0.5 mM DTT, 26% glycerol, 300****mM NaCl, pH 7.9) with a Dounce homogenizer on ice, and incubated for 30****min. Finally the homogenate was centrifuged at 14,000****rpm for 20****min at 4°C, and the nuclear extract was collected from the supernatant.

PI3K was inhibited using LY294002 (Calbiochem) at 20 µM for one hour prior to LPS stimulation. JNK was inhibited using SP600125 (JNK Inhibitor II, Calbiochem) at 20 µM for two hours prior to LPS. SW480 were pre-treated for two hours with the soluble Wnt inhibitor DKK1 at a concentration of 400****ng/mL, prior to the LPS stimulation. To inhibit protein translation we pre-treated the cells with cycloheximide at a concentration of 25 µg/ml for two hours prior LPS stimulation.

### Immunoprecipitation

SW480 or IEC-6 cell line was stimulated with LPS and a total cell lysate was obtained. Total protein was immunoprecipitated using anti-human (total) β-catenin antibody (Sigma-Aldrich) complexed to protein G/plus A. The resulting sample was then analyzed by western blot with an antibody to detect phosphorylation of β-catenin^Ser552^ as well as probed for total β-catenin.

### Western Blot

Protein concentration was analyzed by the Bradford protein assay. Ten percent bisacrilamide gels were used to run the protein samples, and gels were transferred to polyvinylidene fluoride membranes. After washing with tris-buffered saline, membranes were blocked with 5% milk for 30 minutes and then incubated for 1 hour with the primary antibody to detect: GSK3β, P-GSK3β^Ser9^, P-β-catenin^Ser552^, P-β-catenin^Ser675^, AKT, P-AKT^Ser473^ (all antibodies from Cell Signaling), cyclin-D1 (Dako) or β-catenin (Sigma-Aldrich). The above mentioned proteins were then detected by incubation with a secondary antibody conjugated with horseradish peroxidase followed by luminol-based enhanced chemiluminescence HRP substrate. Band densitometry was performed using MyImageAnalysis v1.0 software from Thermo Scientific.

### Statistics

Statistical analyses were performed using GraphPad Prism 5 software. Student *t*-test (2-tailed) was used to detect differences between villin-TLR4 and wild-type mice, for immunohistochemical counts, polyp number counts and western blot densitometry. Results are expressed as mean ± SEM, with a 95% confidence interval. The results were considered statistically significant when the p value was <0.05.

## Results

### TLR4 is Over-expressed by Epithelial Cells in Sporadic Colorectal Cancer

**Figure 1:**
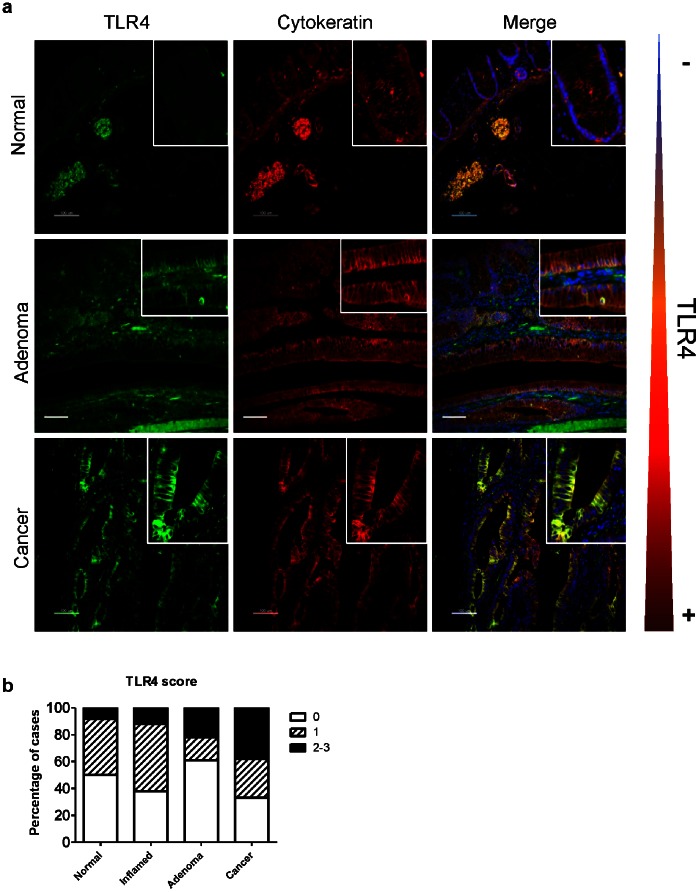
TLR4 is over-expressed by IECs in sporadic colorectal cancer. **a)** Microarrays of human colonic tissues were stained for TLR4 (green), pan-cytokeratin (red) to detect the epithelial compartment, and counterstained with DAPI (blue). Normal tissue, adenoma and cancer. Epithelial TLR4 is best visible in the merged image (yellow) Scale bar: 100 µm. White squares highlight the areas that were magnified. **b)** Graph showing level of TLR4 expression in the colonic epithelium increases from normal to adenoma and cancer in human tissue microarrays. Data represent percentage of cases with TLR4 staining in IECs with score 0 (negative), 1 (low positive), and 2–3 (medium and high positive), for each histopathologic category: normal (n = 12), inflamed (n = 8), adenoma (n = 23), and cancer (n = 52).

TLR4 expression is low in the normal colonic epithelium [23]–[25] (Figure 1). We have previously shown that most cancers arising in ulcerative colitis patients express TLR4. We asked whether TLR4 expression characterized a subset of adenomas and CRCs. To address this question, we examined tissue arrays composed of adenomas and sporadic CRCs for TLR4 expression by immunofluorescent staining (Figure 1a). Using a TLR4 scoring system (negative = 0, low positive = 1, positive = 2, high positive = 3), we demonstrate that 22% of adenomas and 38% of sporadic CRCs show increased expression of TLR4 (score 2–3) compared to 8% of normal tissues (8% score 2–3, 92% score 0–1) (Figure 1b). In addition, not only does TLR4 expression increase in the progression from normal to cancer, but we also observed a change in localization of TLR4 in the cell from predominantly in the basolateral aspect in normal tissue and in adenomas, to the apical and basolateral surface in a subset of sporadic CRCs. These data suggest that increased TLR4 expression occurs in both precancerous colonic adenomas and in colon cancer, prompting further investigation into the role of TLR4 in the transition from normal to neoplasia.

### TLR4 Activation Drives Expansion of Crypt Epithelial Cells

**Figure 2:**
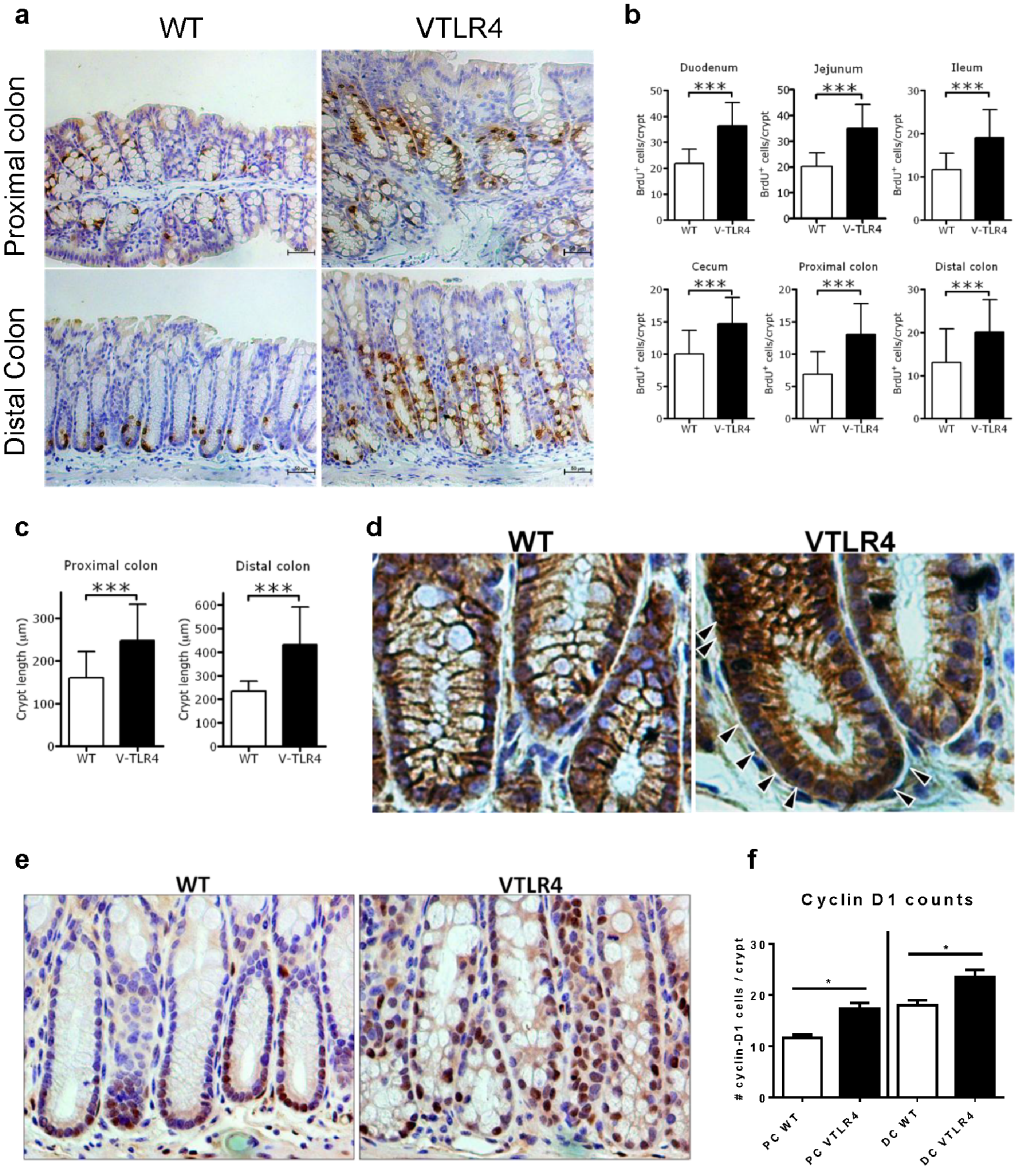
TLR4 increases colonic cell proliferation. **a)** BrdU immunostaining of the colon (40x). Villin-TLR4 mice (VTLR4) show increased cell proliferation in proximal and distal colon, compared to wild-type (WT) mice. **b)** Graph of BrdU positive cells per crypt. Villin-TLR4 mice show a significant increase of proliferating cells compared to WT mice (***p<0.05). **c)** Graph of crypt length of the colon. In villin-TLR4 mice the intestinal crypts are taller (approx. 300–400 µm) than the WT counterparts (approx. 200 µm). **d)** Villin-TLR4 mice showed greater cytoplasmic and nuclear β-catenin compared to WT mice in which the β-catenin is mainly expressed in the cell membrane (brown). Arrows highlight nuclear staining. **e)** and **f)** Expression of cyclin-D1 was also increased in villin-TLR4 mice compared with WT mice.

Given that TLR4 expression is increased in the epithelial compartment of adenomas and cancer, we chose to model this observation in mice. Villin-TLR4 mice express a TLR4 transgene under the control of a villin promoter that directs gene expression in epithelial cells from the crypt to the top of the villi [26]. Expression of the transgene has been confirmed throughout the epithelium of the gastrointestinal tract from the duodenum to the colon [21]. We asked whether expression of TLR4 alters cellular proliferation in the intestine by short-term injection of 5-bromo-2′-deoxyuridine (BrdU) followed by immunostaining against BrdU (Figure 2a). We observed significantly more BrdU positive cells along the crypt length of villin-TLR4 compared to wild-type mice. When compared to wild-type mice, the proliferative index of villin-TLR4 mice was significantly greater in each segment of the small and large intestine (Figure 2b). This increased proliferation in intestinal epithelial cells (IEC) resulted in significantly longer colonic crypts in the villin-TLR4 mice than in wild-type mice especially in the distal colon, the site of most colon cancers in humans (Figure 2c). Additionally, we examined β-catenin expression and activation of cyclin-D1, a Wnt target gene associated with cellular proliferation, and found that expression of nuclear β-catenin and cyclin-D1 positive nuclei were increased along the colonic crypt axis in villin-TLR4 mice compared with wild-type mice (Figure 2d, e, f). These data demonstrate that TLR4 signaling activates a program of proliferation in IECs.

The finding of TLR4-mediated proliferation and increased crypt length led us to ask whether activation of TLR4 changed the dynamics of crypt cell propagation. Lgr5 has been identified as a Wnt target gene and a marker of intestinal stem cells [27]. We asked whether TLR4 regulates epithelial proliferation manifested by Lgr5-positive cells. To answer this question we crossed villin-TLR4 mice to Lgr5-EGFP mice (B6.129P2-Lgr5^tm1(cre/ESR1)Cle^/J ), which express EGFP under the Lgr5 promoter, and quantified the number of Lgr5+ cells per crypt. We found that while Lgr5 positive cells are located only at the base of the crypts in wild-type mice, there is a dramatic increase in Lgr5+ cells along the length of crypts of villin-TLR4 mice, especially in the distal colon (Figure S3). These data indicate that TLR4 is linked to increased epithelial proliferation in the intestine.

### TLR4 Induces Colonic Tumors in Response to Genotoxic Stress

**Figure 3:**
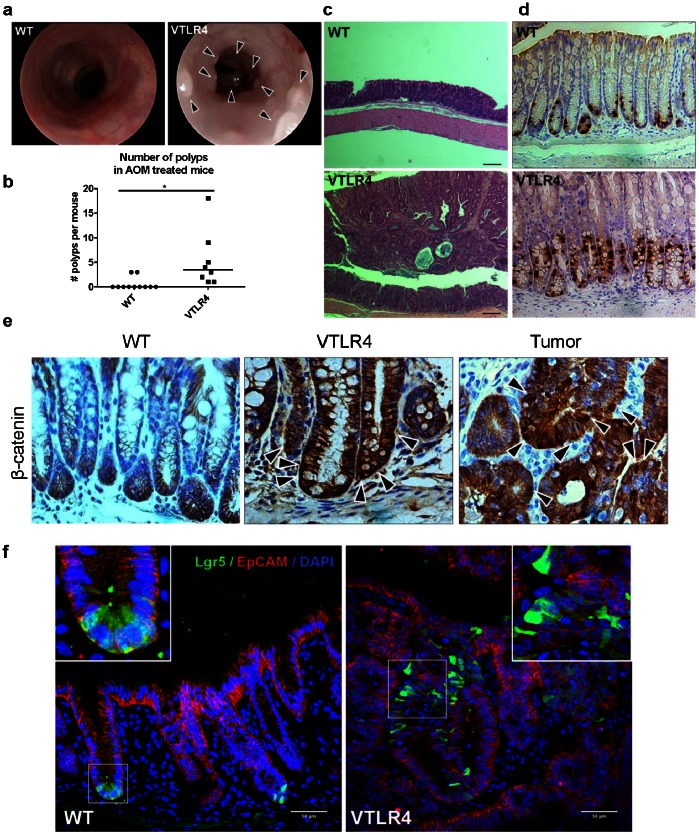
TLR4 promotes colonic neoplasia in response to genotoxic stress. **a)** Polyp development in AOM-treated mice was monitored by colonoscopy. Pictures were taken at week 17 after treatment, right before sacrifice. **b)** In distal colon, Villin-TLR4 mice develop a higher number of polyps than WT mice. **c)** Hematoxylin & eosin staining showing the histology of WT and VTLR4 (colon polyp and surrounding) after AOM (4x). **d)** BrdU staining in AOM-treated mice (20x). Villin-TLR4 mice show increased cell proliferation compared with WT mice. **e)** β-catenin immunostaining in AOM-treated mice (40x). Villin-TLR4 mice had increased expression of active β-catenin (cytoplasmic and nuclear) in the tumor and the surrounding normal tissue, compared to WT mice. **f)** Immunofluorescent staining of WT and villin-TLR4 Lgr5-EGFP reporter mice, treated with AOM. Villin-TLR4 tumors in the distal colon were characterized by Lgr5+ cells (green). Counterstaining by EpCAM (red, IECs) and DAPI (blue, nuclei) (40x). White squares highlight magnified areas.

Given that certain adenomas and CRCs over-express TLR4, we asked whether TLR4 functioned as a tumor promoter. We used the mutagenic agent azoxymethane (AOM), which has been shown to induce colonic tumors in certain strains of mice [28], [29]. C57BL/6 mice, the background strain for our TLR4 transgenic animals, are resistant to AOM-induced tumors [30], [31]. Therefore, we asked whether AOM alone was sufficient to induce tumors in villin-TLR4 mice. In wild-type mice, AOM treatment rarely resulted in tumors (Figure 3a). By contrast, AOM induced robust colonic tumorigenesis in villin-TLR4 mice (Figure 3a). Furthermore, villin-TLR4 mice developed a significantly higher number of polyps in the distal colon than WT mice (VTLR4: median = 3.5 tumors, range 1–18, n = 8 *vs* WT: median = 0 tumors, range 0–3, n = 11; *p = 0.014) (Figure 3b). Histologically, these tumors ranged from low-grade to high-grade dysplasia and carcinoma *in situ* similar to adenomatous polyps in humans (Figure 3c). Histologic scoring of villin-TLR4 and wild-type mice before and after AOM did not reveal any acute inflammation (Figure S4). Tumors arising in villin-TLR4 mice are characterized by intense nuclear β-catenin staining (Figure 3e) and a population of Lgr5-positive cells, a marker of cancer stem cells (Figure 3f) [32]. Both the tumors and surrounding tissue also had a dramatic increase in proliferating cells in villin-TLR4 mice that was not seen in wild-type mice (Figure 3d). Thus, epithelial TLR4 expression induces colonic neoplasia and β-catenin activation in the absence of inflammation.

### Villin-TLR4 mice Develop Spontaneous Duodenal Adenomas

**Figure 4:**
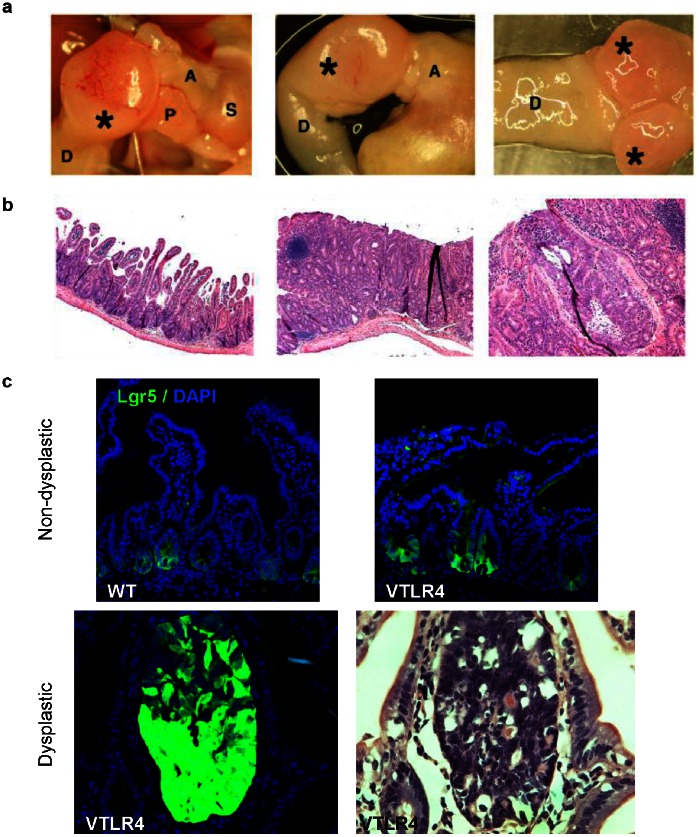
Villin-TLR4 develop spontaneous duodenal adenomas. **a)** Macroscopic images of spontaneous duodenal polyps in villin-TLR4 mice. Asterisks indicate tumors. S: Stomach, A: antrum, P: Pancreas, D: duodenum. Pictures show from left to right: anatomical view, isolated tumor (*) with stomach (A) and normal duodenum (D), luminal view of the tumor (*). **b**) Histological pictures of the duodenum. Some of the tumor contains high-grade dysplasia; left to right: duodenal mucosa (10x), adenoma (10x), high-grade dysplasia (20x). **c)** Immunostaining for Lgr5 in duodenal tissue. Top panel: normal duodenum in a wild-type Lgr5-EGFP mouse; normal duodenum in a villin-TLR4 Lgr5-EGFP mouse; Bottom panel: duodenal adenoma in a villin-TLR4 mouse with infiltrate of Lgr5+ cells; H&E staining of the villin-TLR4 duodenal adenoma (40x). Lgr5 is shown in green and counterstained in blue with nuclear DAPI (40X).

Given the potent tumor-prone phenotype seen in the colon of villin-TLR4 mice, we examined villin-TLR4 mice for the spontaneous development of tumors. Villin-TLR4 mice developed duodenal adenomas starting from 12 weeks of age (Figure 4a). Microscopically aberrant crypt foci appeared in the duodenum after 9 weeks of age. Although most tumors were characterized by low-grade dysplasia, some of the duodenal tumors had foci of high-grade dysplasia (Figure 4b). Duodenal crypts from villin-TLR4 mice had an increase in Lgr5+ cells compared to wild-type mice (Figure 4c). We followed the villin-TLR4 mice up to 64 weeks and none developed carcinomas. Staining for β-catenin revealed nuclear staining in the dysplastic areas and intense cytoplasmic staining even in the histologically normal duodenal mucosa (Figure S5a). In addition, cyclin-D1 was also increased in the normal and dysplastic areas in the duodenum of villin-TLR4 mice compared to wild-type mice (Figure S5b). These findings support our hypothesis that activation of TLR4 in IEC promotes the development of intestinal neoplasia through β-catenin pathway activation.

### TLR4 Signaling Activates the β-catenin Pathway in Intestinal Epithelial Cells

**Figure 5:**
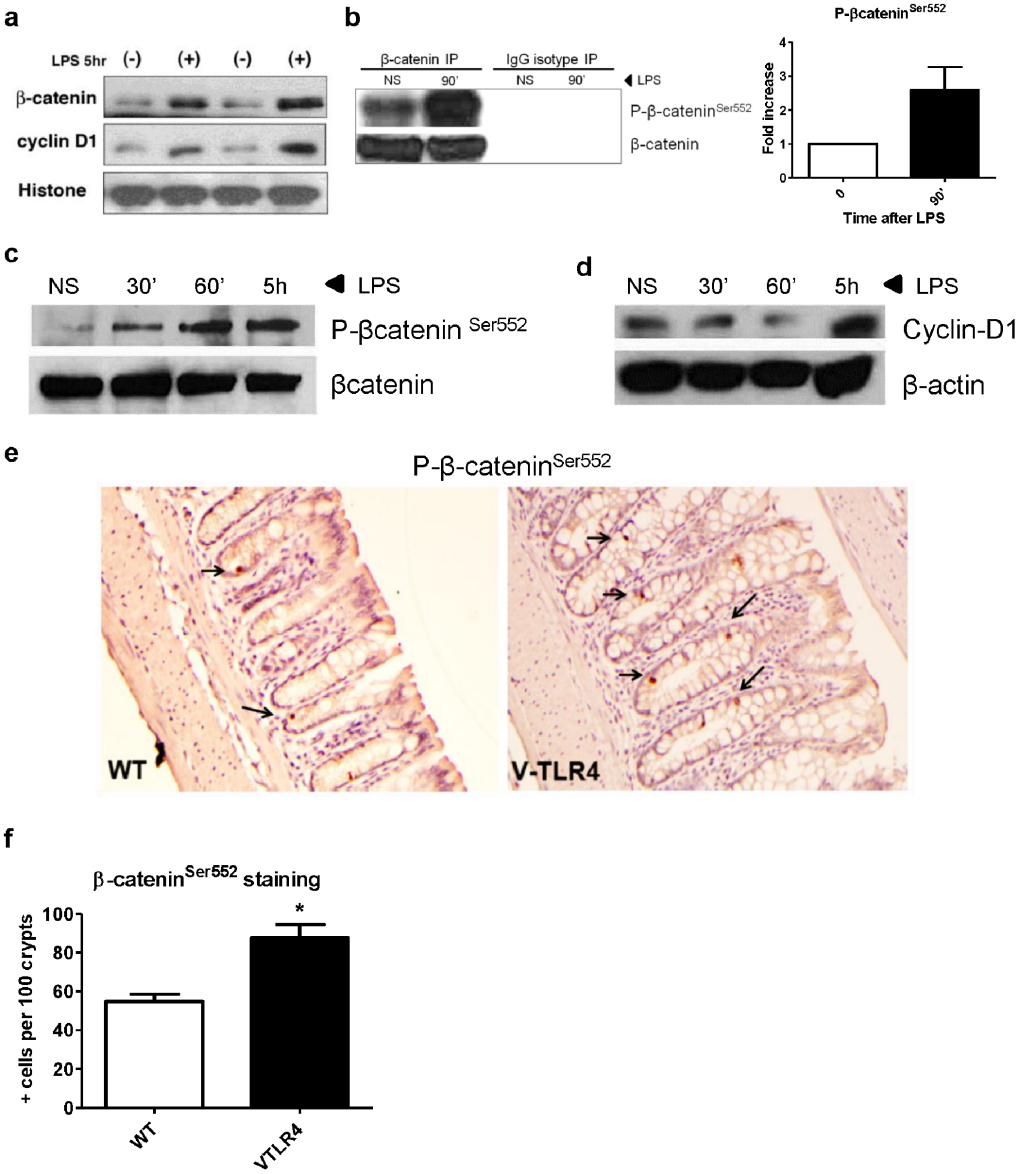
TLR4 activates the β-catenin pathway. **a)** Western blot of IECs (SW480) stimulated with the TLR4 ligand LPS (+). In vitro LPS-stimulated cells have increased expression of nuclear β-catenin and cyclin-D1 compared to non-stimulated cells (-) (Two replicates shown). Histone 1 expression used as loading control for nuclear lysates. **b)** Western blot of SW480 total cell extracts immunoprecipitated for total β-catenin, and immunoblotted for P-β-catenin^Ser552^. P-β-catenin^Ser552^ increases 1.5h after incubation with LPS. Graph on the right shows band densitometry analysis. **c)** Western blot analysis shows that IEC-6 cells respond to LPS inducing P-β-catenin^Ser552^ with β-catenin as loading control, and **d)** cyclin-D1 expression with β-actin used as loading control. **e)** Immunostaining for P-βcatenin^Ser552^ (black arrows) in AOM-DSS villin-TLR4 and WT mice (20x). **f)** Graph of counts of P-βcatenin^Ser552^ positive cells per 100 colon crypts. Villin-TLR4 mice treated with AOM-DSS have increased P-βcatenin^Ser552^ in the colon epithelium compared with their WT counterparts.

β-catenin plays a central role in intestinal proliferation and neoplastic transformation. *In vivo*, we have shown that TLR4 drives epithelial β-catenin activation and Lgr5+ cell expansion at baseline and in response to genotoxic stress. We now wished to understand whether activation of β-catenin by TLR4 occurred in a cell autonomous fashion. To address this question, we used model colonic epithelial cells. SW480 cells harbor a mutated APC gene [33]–[35] and mimic the most common mutation found in human sporadic colon cancer. IEC-6 is a non-transformed intestinal cell line that expresses a wild-type APC gene [36], [37]. Both SW480 and IEC-6 cells express TLR4 and respond to LPS stimulation, as measured by NF-κB induction [38], [39] (Figure S6). We found that stimulation with LPS induced nuclear localization of β-catenin and cyclin-D1 compared with unstimulated cells in both SW480 (Figure 5a) and IEC-6 cells (Figure 5c, b).

Phosphorylation of β-catenin at serine 552 (β-catenin^Ser552^) and serine 675 (β-catenin^Ser675^) is associated with nuclear import of β-catenin, a hallmark of its active state [40]. We found that LPS induced both of these phosphorylation events in IECs within 90 minutes providing further biochemical evidence of β-catenin activation by TLR4 (Figure 5b, Figure S7). Thus, TLR4 activates β-catenin in IECs and this effect is observed both in APC wild-type cells (IEC-6) and APC mutated cells (SW480). Finally, we asked whether TLR4 activation *in vivo* was associated with phosphorylation of β-catenin^Ser552^. This phosphorylation event has been linked with stem cell activation in the setting of inflammatory neoplasia [22]. In mice treated with AOM and dextran sodium sulfate (DSS), we found a significant increase in crypt epithelial cells with phosphorylated β-catenin^Ser552^ in non-dysplastic colonic epithelium of villin-TLR4 mice compared to wild-type mice (VTLR4: 87.6±6.9, n = 10; *vs* WT: 54.8±3.9, n = 8; *p = 0.0014) (Figure 5e, f).

### TLR4 Activates the β-catenin Pathway in a PI3K-dependent Fashion

**Figure 6:**
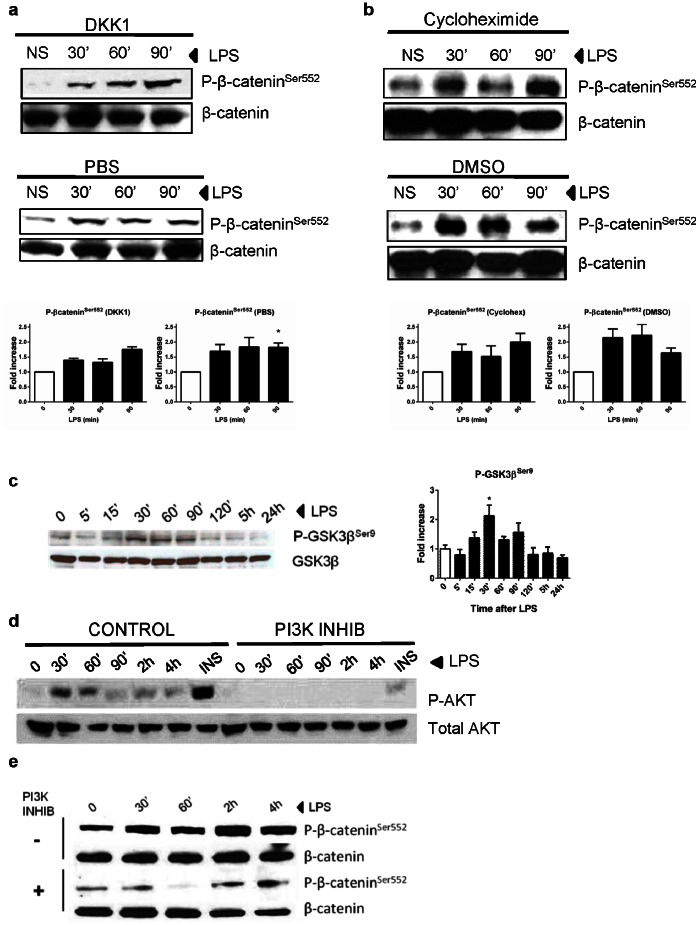
TLR4 activates the Wnt/β-catenin pathway in a PI3K-dependent fashion. **a)** Western blot showing the effect of the Wnt inhibitor DKK1 in SW480 cells treated prior of addition of LPS. DKK1 pre-treatment (top panel) does not change TLR4-mediated β-catenin activation (P-β-catenin^Ser552^), compared to untreated cells (mid panel). Total β-catenin is shown as the loading control. Bottom panel shows densitometry analysis of 3 blots (*p<0.05). **b)** Western blot showing the effect of the inhibitor of protein translation cycloheximide in pre-treated SW480, prior LPS stimulation. Cycloheximide does not change TLR4-mediated β-catenin activation (P-β-catenin^Ser552^) (top panel), compared to untreated cells (mid panel). Total β-catenin is shown as the loading control. Bottom panel shows densitometry analysis of 3 blots. **c)** Western blot in IECs (SW480) for P-GSK3β^Ser9^. 30–90 minutes after LPS stimulation, there is an increase in P-GSK3β^Ser9^, with peak activation at 30 minutes (*p = 0.042, n = 3). Graph on the right shows densitometry data of 3 independent experiments with similar results. Total GSK3β used as loading control. **d)** Western blots of SW480 stimulated with LPS at different time-points, show an increase in phosphorylation of P-AKT^Ser473^ at 30 minutes, which is inhibited by the PI3K inhibitor LY294002. Insulin (INS) was used as a positive control for AKT activation. Total AKT used as loading control. **e)** Western blot in SW480 cells pre-treated with LY294002, a PI3K inhibitor. After LPS stimulation, there is a decrease in P-β-catenin^Ser552^ in cells pre-treated with LY294002. Total β-catenin used as loading control.

The molecular mechanism that links TLR4 and β-catenin is currently unknown. We first determined if Wnt-receptor interaction was required for β-catenin activation. Soluble dickkopf-1 (DKK1) is an antagonist of Wnt signaling that binds to LRP5/6 and prevents its interaction with Wnt-Frizzled complexes [41]–[43]. We stimulated colonic IECs with LPS in the presence of DKK1 to determine the dependence of Wnt signaling on TLR4-induced β-catenin. We found that TLR4 activation by LPS continued to induce phosphorylation of β-catenin^Ser552^ in the presence of DKK1 (Figure 6a), suggesting that TLR4-dependent β-catenin phosphorylation was not dependent on induction of Wnt secretion.

Cytokines such as IL-1β have been shown to activate the Wnt pathway in a paracrine fashion [44], [45]. Therefore, we asked if new protein synthesis was required for TLR4-dependent activation of β-catenin. We found that treatment of SW480 cells with cycloheximide did not prevent TLR4-induced phosphorylation of β-catenin^Ser552^ suggesting that this occurred through a signal transduction mechanism (Figure 6b). Similarly, inhibition of gene transcription with actinomycin-D had no effect on TLR4-mediated β-catenin activation (data not shown).

To further elucidate the signaling pathway that links TLR4 to β-catenin phosphorylation and nuclear translocation, we analyzed whether TLR4 activation resulted in GSK3β phosphorylation. GSK3β phosphorylation at serine 9 is associated with protection of β-catenin from proteosomal degradation [46]. In response to LPS stimulation, GSK3β is rapidly phosphorylated at serine 9 in both SW480 (Figure 6c) and IEC-6 cells (Figure S8).

We then used a bioinformatics approach to link TLR4 to β−catenin. GeneGo putative pathway modeling indicated that β−catenin phosphorylation by TLR4 could occur through PI3K activation. It is well known that PI3K activates AKT in mammalian cells [47]–[49]. In addition, TLR4 has been shown to activate the kinase AKT in alveolar macrophages and CRC cells [50], [51] downstream of MyD88 [52]. However, this link has never been studied in the colon. Phosphorylation of β-catenin^Ser552^ has been linked to PI3K activation *in vivo* and *in vitro* [22], [53]. TLR4 activation of SW480 cells led to potent phosphorylation of AKT (Figure 6d). LY294002, a chemical inhibitor that blocks PI3K activity, prevented TLR4-dependent phosphorylation of β-catenin^Ser552^ (Figure 6e). As a control for these experiments, we asked whether inhibition of JNK blocked TLR4-dependent phosphorylation of β-catenin^Ser552^. Inhibiting JNK with SP600125 did not inhibit TLR4 -dependent β-catenin^Ser552^ phosphorylation (Figure S9). These data support a model in which TLR4 signaling in the epithelium activates the β-catenin pathway through PI3K and is not dependent on Wnt secretion or new protein synthesis.

## Discussion

**Figure 7:**
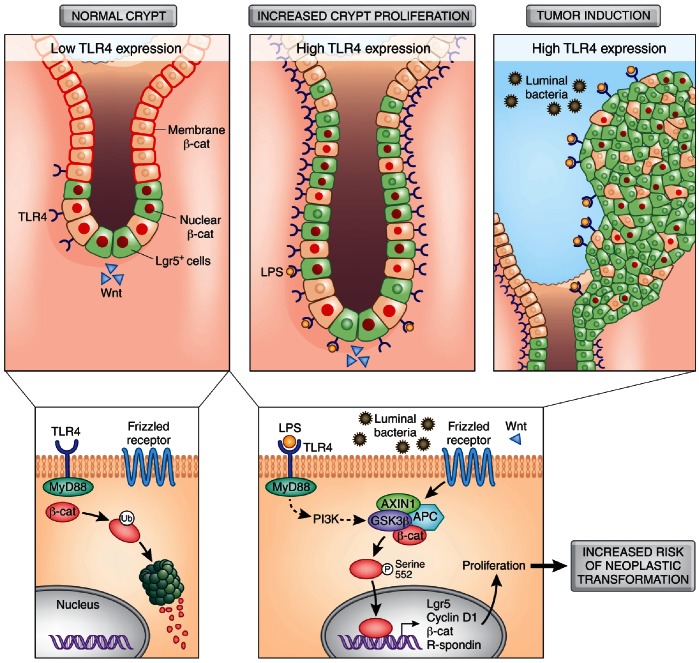
Model for TLR4 driven tumorigenesis. TLR4 promotes neoplasia through activation of the β-catenin pathway. Under normal conditions, TLR4 expression is low in the colon crypts, with few proliferating cells at their base expressing Lgr5 (green cells) and nuclear β-catenin. The majority of the cells along the crypt have β-catenin undergoing proteosomal degradation (membrane β-catenin). TLR4 activation results in activation of β-catenin, increased expression of Lgr5, and increased proliferation. In a third of CRCs, TLR4 is over-expressed, promoting tonic stimulation of cell proliferation and an expansion of cancer cells.

In the current study, we demonstrate the importance of TLR4, an innate immune receptor, in intestinal tumorigenesis and provide evidence that TLR4 acts as a potent tumor promoter (Figure 7). We demonstrate that in humans, almost 40% of sporadic CRC and 20% of colon adenomas over-express TLR4 (Figure 1). As a proof of principle, mice that over-express TLR4 in the intestinal epithelium have a much lower threshold for developing intestinal tumors. These tumors occur in the absence of overt inflammation suggesting that persistent stimulation of TLR4 is detrimental to the host’s gastrointestinal epithelium and that it primes the development of intestinal neoplasia. This TLR4-mediated neoplastic drive is exacerbated in the colonic epithelium by the addition of the mutagen AOM. Experimentally, we show that TLR4 activates the central pathway of proliferation and regeneration in the intestine–the β-catenin pathway. When one considers the stimulus provided by luminal bacteria, it is possible to imagine how subtle perturbations in TLR4 expression can result in activation of oncogenic pathways and the resulting neoplastic transformation. These data permit us to speculate on the novel possibility that the TLR4 status of an adenoma or cancer could be used to design targeted strategies such as TLR4 antagonists to prevent or treat cancer.

Accumulating evidence points to TLR4 as a participant in CRC; the mechanism by which this occurs has not been elucidated in its entirety, but the present study supports a role for the canonical Wnt pathway and β-catenin. To explore how TLR4 might participate in colonic tumor development, we took a multifaceted approach. Given that human tumors over-express TLR4 in the epithelium, we mimicked this using the villin promoter to drive expression of a TLR4 transgene. Although all experimental models of colonic neoplasia are fundamentally imperfect, we feel that over-expression of TLR4 offers insight into whether TLR4 has a distinct role as a tumor initiator and promoter. It is also different from the customary approach of using knock-out mice, which does not represent what occurs in human colon cancers. The most striking phenotypic observation in villin-TLR4 mice was the increase in epithelial proliferation and crypt height that was apparent when compared with WT controls. Lgr5 has been used to study tumor promoting signals in intestinal stem cells [27], [54]–[56]. Because of advances in the characterization of stem cells in the intestine, we crossed our villin-TLR4 mice to Lgr5-EGFP mice so that we could evaluate whether the crypt expansion was due to an increase in Lgr5-expressing cells. Here we provide data that dysregulated TLR4 signaling can alter the dynamics of Lgr5+ cells in the intestine. In spite of the variegated expression of the Lgr5-EGFP knock-in gene, the colon crypts that expressed EGFP showed a large increase in the number of Lgr5+ cells per crypt in villin-TLR4 mice that was not seen in WT mice. In addition, we found that villin-TLR4 mice have an increase in cell proliferation as well as an increase in Lgr5+ cells in the duodenum (Figure 4). Neal et al. recently reported that TLR4 is expressed in intestinal stem cells [57]. In their study, they showed that intestinal stem cells (Lgr5+) from WT mice had reduced proliferation compared to TLR4 knockout mice; they do not describe what happens in the colon. Our study is the first that looks at the Lgr5+ stem cell population in a model where TLR4 is selectively over-expressed in the intestinal epithelium. We compared the colon and duodenum of wild-type mice *versus* villin-TLR4 mice and found a clear increase in Lgr5+ cells in mice with increased expression of TLR4 in the epithelium. It is difficult to compare our approach in which a transgenic approach is used to increase epithelial expression of TLR4 to a whole animal knock-out of TLR4. In addition, our villin-TLR4 mice develop duodenal adenomas with Lgr5+ tumor cells, even in the absence of a genotoxic agent, supporting the idea that TLR4 activates proliferation. Moreover, in the presence of the carcinogen AOM, TLR4 over-expression was sufficient to cause colonic tumors that were not seen in WT mice [31]. These data allow us to conclude that TLR4 signaling in the intestinal epithelium provides a potent proliferative drive that culminates in neoplastic transformation in the presence of added mutagenic stimuli.

With this as a backdrop, we asked the provocative question: can TLR4 directly activate β-catenin signaling? We present biochemical and functional data to support the notion that TLR4 signaling results in downstream cell autonomous β-catenin activation and expression of Wnt target genes *in vivo* and *in vitro*. There are likely several pathways that connect TLR4 to β-catenin activation; we have mechanistically traced the pathway from TLR4 to activation of PI3K/AKT with subsequent phosphorylation of GSK3β and β-catenin. The intermediary step of TLR4-mediated PI3K activation complements data demonstrating the role of PI3K in stem cell activation in colitis-associated neoplasia [22]. The latter study did not explore targets upstream of PI3K in inflammatory neoplasia. Our study connects TLR4 to PI3K and β-catenin. We have previously shown that the AOM-DSS model of colitis-associated cancer is characterized by increased epithelial TLR4 expression; thus it is possible that TLR4 is one of the mediators of PI3K activation seen in CAC [9]. EGFR can also trans-activate PI3K [58], and physically interact with β-catenin [59], [60]. Our group has previously found that TLR4 stimulates EGFR phosphorylation and expression of EGFR ligands, amphiregulin, and epiregulin [9], [38], [61]. Therefore, TLR4 might activate a network of signals that culminate with β-catenin activation. In the present manuscript, we consistently show clear activation of β-catenin through TLR4 signaling, suggesting that targeting TLR4 could have a broad effect on oncogenic signaling pathways.

Our data provide complementary mechanistic insight into earlier studies which have shown that MyD88, an adaptor protein used by TLR4 as well as by other cell receptors, was required for the growth of APC-dependent tumors [62], [63]. These studies demonstrated the importance of MyD88 in the up-regulation of the c-myc oncogene through post-translational modification by ERK, with smaller intestinal tumors identified in MyD88^−/−^×APC^min/+^ mice. Lee *et al*. also suggest that MyD88 signaling does not regulate β-catenin in their comparison of WT to MyD88-deficient mice [62]. Our study demonstrates that in the setting of intact MyD88 signaling, TLR4 is an important promoter of tumorigenesis, and activates β-catenin. We show the increased activity of the β-catenin pathway not only in our villin-TLR4 mouse model, but also in intestinal epithelial cell lines. These observations further substantiate the evolving awareness that MyD88^−/−^ and TLR4^−/−^ mice behave differently in response to oncogenic stimuli. Illustrative of this point, TLR4^−/−^ mice are resistant to AOM-DSS induced tumors [9]; whereas MyD88^−/−^ are more susceptible to intestinal neoplasia with AOM-DSS. As MyD88 serves as an adaptor to several different receptors (not only TLRs), we propose that the oncogenic signaling via TLR4 in the setting of normal MyD88 differs from the signaling pathways driving tumorigenesis in MyD88^−/−^ mice. In terms of translation to the clinical realm, MyD88 is not an appealing target because of its role as an adaptor for multiple receptors that could result in broad immunological effects; modulation of the TLR4 molecule represents a more favorable clinical strategy. In preclinical models, we and others have shown that blocking TLR4 could prevent colonic tumor development or reduce metastatic tumor burden [9], [14], [64]. These data point to TLR4 signaling as a point of intervention in future clinical studies.

In contrast to our results, a study by Sodhi *et al.* suggested that TLR4 inhibits β-catenin and impairs enterocyte proliferation in the small intestine of neonatal mice with experimental necrotizing enterocolitis [65]. This effect was not seen in the colon of the same neonatal mice nor was it found anywhere in the intestine of adult mice. We show here that TLR4 over-expression induces β-catenin phosphorylation in the colon *in vivo*, and we have confirmed our findings *in vitro* using two different cells lines. We have previously shown that TLR4 signaling in the colon induces epithelial proliferation and protection against apoptosis [66], [67]. Together these data suggest that the consequences of TLR4 signaling evolve during development from the neonatal period to adulthood coincident with the anatomical specialization of the small bowel and the colon.

In conclusion, we believe that TLR4 is a potent tumor promoter in the intestine. TLR4 activates β-catenin through an intracellular signaling pathway leading to increased cell proliferation and neoplasia. The results of our work have broad implications for targeting TLRs to prevent or treat colon cancer and for the development of biomarkers in patients with colonic adenomas.

## Supporting Information

Figure S1 **Tumorigenesis was chemically induced with a total of 6 doses of AOM, administered weekly, at a concentration of 14.8 mg/kg.** After week 17 the mice were sacrificed and tissue was collected.
(TIF)

Figure S2 **Negative control omitting the primary antibody for the immunofluorescence performed in** **Figure 1** **.** TLR4 (green), Cytokeratin (red), DAPI (blue). Scale bar = 100 µm.
(TIF)

Figure S3 Formalin-fixed paraffin embedded tissues from **(a)** WT Lgr5-EGFP mice (B6.129P2-Lgr5^tm1(cre/ESR1)Cle^/J) and **(b)** villin-TLR4 Lgr5-EGFP mice (villin-TLR4×B6.129P2-Lgr5^tm1(cre/ESR1)Cle^/J) were cut to detect Lgr5 expression by immunofluorescent staining. We found that in Lgr5+ crypts from the distal colon, Lgr5+ cells were restricted to the bottom of the colonic crypt in WT Lgr5-EGFP mice while Lgr5+ cells in the positive crypts of villin-TLR4 Lgr5-EGFP mice (villin-TLR4×B6.129P2-Lgr5^tm1(cre/ESR1)Cle^/J) were present throughout the height of the crypt. Lgr5+ cells are shown in green and nuclear counterstaining with DAPI in blue (63x). **c)** Negative control without primary antibody (anti-EGFP) ruled out background staining from the Lgr5-EGFP staining experiment. **d)** Graph of Lgr5+ cells in crypts with Lgr5-EGFP expression. Villin-TLR4 mice show a higher amount of Lgr5+ cells than WT mice, in both the proximal (WT: 1.95±0.54 n = 20; VTLR4: 7.57±0.57 n = 7; *p<0.0001) and distal colon (WT: 3.55±0.37 n = 20; VTLR4: 17.30±1.426 n = 20; *p<0.0001).
(TIF)

Figure S4 **a)** Villin-TLR4 (VTLR4) mice do not have baseline inflammation compared to their wild-type (WT) littermates. However VTLR4 show longer colonic crypts compared to WT mice. Both images were taken under the same microscope objective (20x). **b)** Inflammation was scored in Villin-TLR4 and wild-type mice at baseline and after AOM treatment. There were no significant differences between the two strains neither at baseline (WT: 1.277±0.1468 N = 3, VTLR4: 1.223±0.3359 N = 3, p = 0.320) nor after AOM treatment (WT: 0.6250±0.3146 N = 4, VTLR4: 2.134±0.4334 N = 5, p = 0.503). The inflammatory score can go from 0 to 20, and has into account: crypt damage (0–4), acute inflammation (0–4), edema (0–3), necrosis/ulceration (0–3), chronic inflammation (0–3) and epithelial regeneration (0–3).
(TIF)

Figure S5 **a)** Spontaneous duodenal adenomas in villin-TLR4 mice stain for nuclear and cytoplasmic β-catenin (brown), by immunohistochemistry. **b)** These adenomas are also characterized by cyclin-D1 positive epithelial cells (brown).
(TIF)

Figure S6 Graphs showing NFκB induction after LPS stimulation in **a)** SW480 cells and **b)** IEC-6 cells, measured by NFκB reporter luciferase assay.
(TIF)

Figure S7 **a)** Western blot analysis in SW480 nuclear fractions harvested 90 minutes after LPS stimulation. LPS-treated cells show increased phosphorylation of β-catenin at Ser675 (P-β-catenin^Ser675^) in the nuclear fraction. Conversely, the amount of P-β-catenin^Ser675^ decreases in the cytoplasmic fraction, suggesting β-catenin translocation into the nucleus; **b)** and **c)** show the densitometry analysis of both cellular fractions.
(TIF)

Figure S8 **IEC-6 cells respond to LPS with phosphorylation of GSK-3β at Serine 9 (P-GSK3β^Ser9^).** Total GSK3β is shown as loading control.
(TIF)

Figure S9 **TLR4-mediated β-catenin activation is not affected by JNK signaling in intestinal epithelial cells.** IEC-6 cells stimulated with LPS show an increase of β-catenin^Ser552^ phosphorylation in the absence **(a)** or presence of the SP600125 JNK inhibitor **(b).**
(TIFF)
