# Supplementary figures and images for: TLR4 Activates the β-catenin Pathway to Cause Intestinal Neoplasia
Source: PLoS One. 2013 May 14;8(5):e63298. doi: 10.1371/journal.pone.0063298 (PMC3653932; doi:10.1371/journal.pone.0063298)

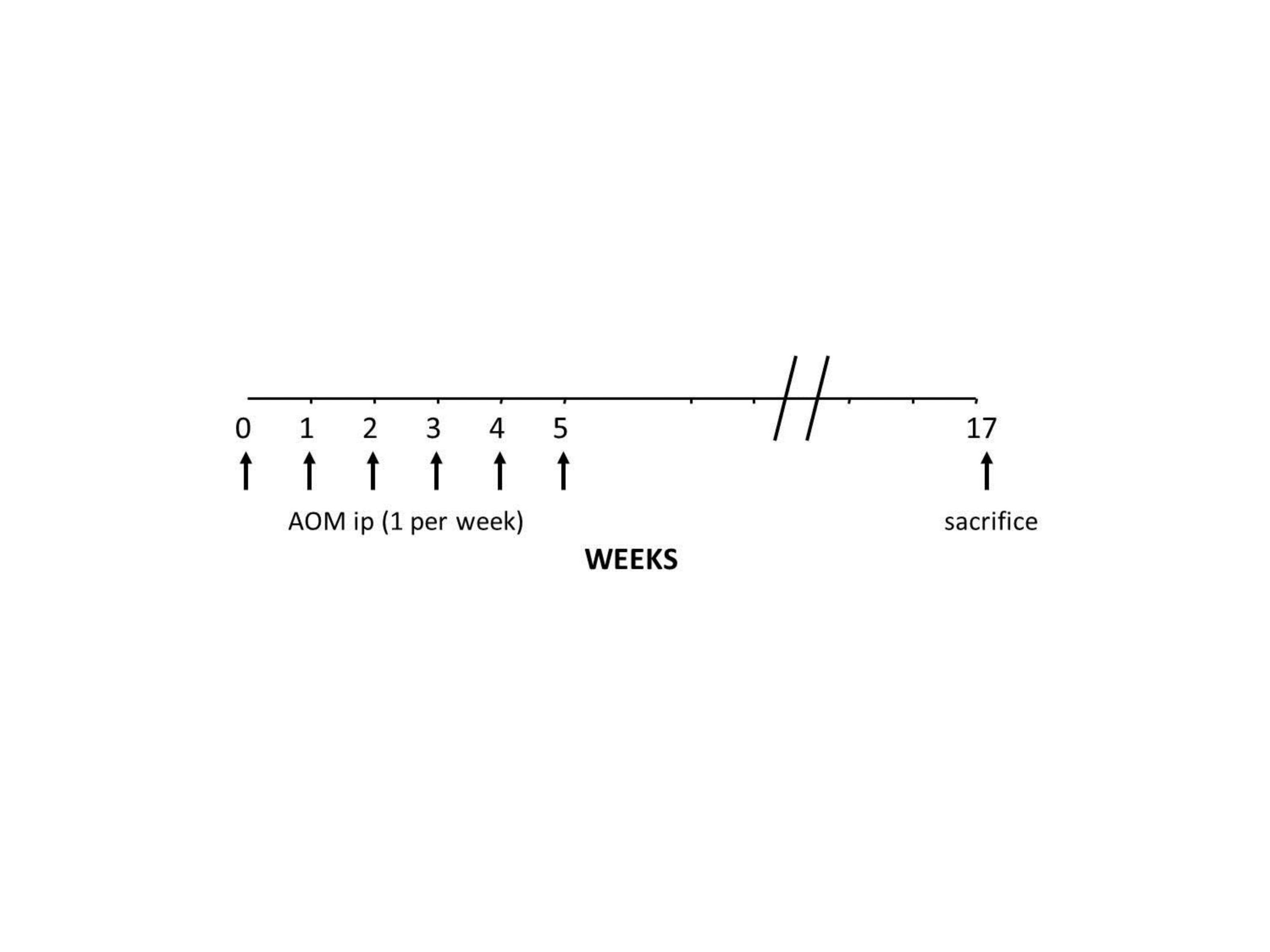

Supplement: Figure S1 — Tumorigenesis was chemically induced with a total of 6 doses of AOM, administered weekly, at a concentration of 14.8 mg/kg. After week 17 the mice were sacrificed and tissue was collected. (TIF) [file pone.0063298.s001.tif]

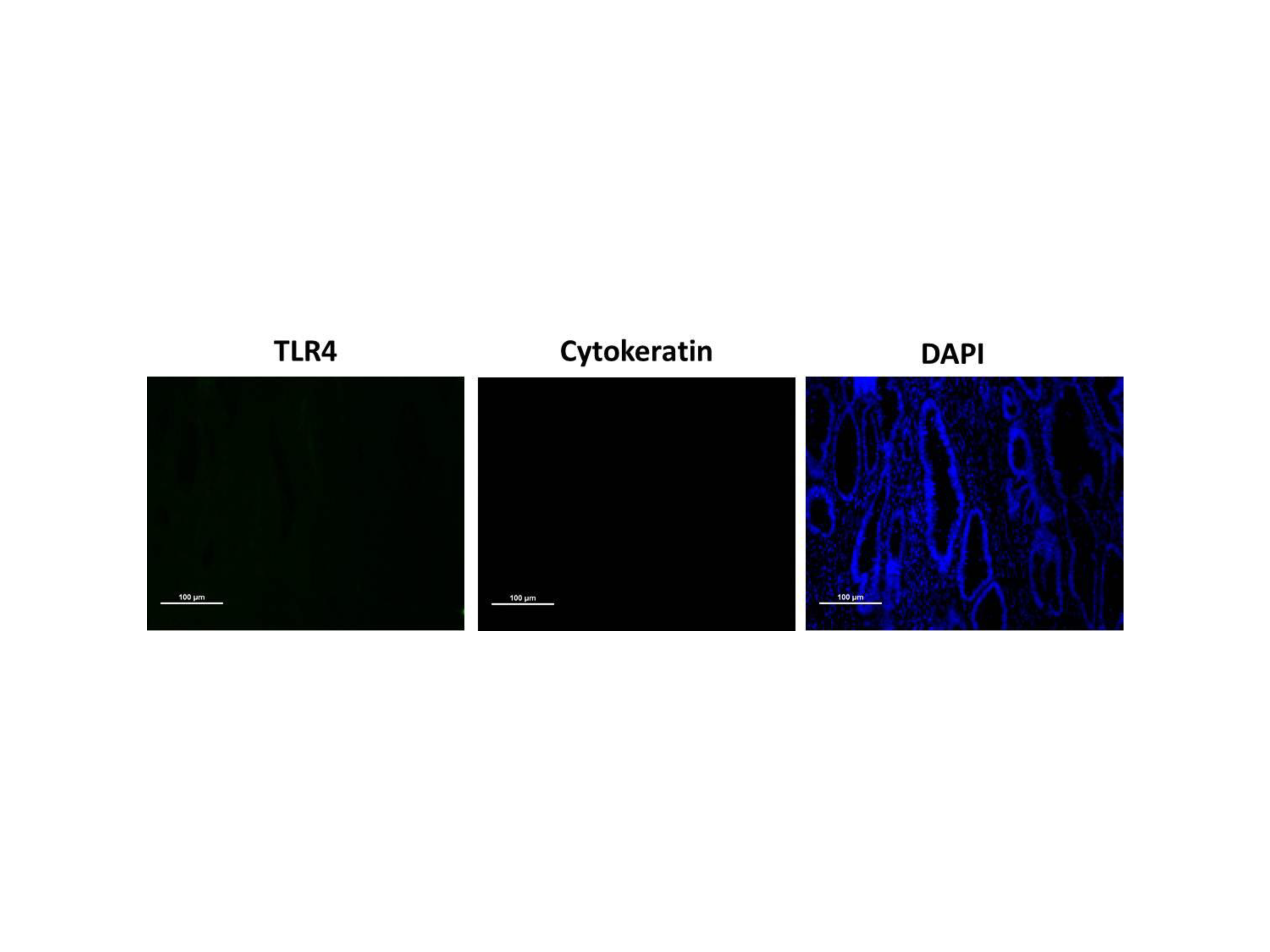

Supplement: Figure S2 — Negative control omitting the primary antibody for the immunofluorescence performed in Figure 1 . TLR4 (green), Cytokeratin (red), DAPI (blue). Scale bar = 100 µm. (TIF) [file pone.0063298.s002.tif]

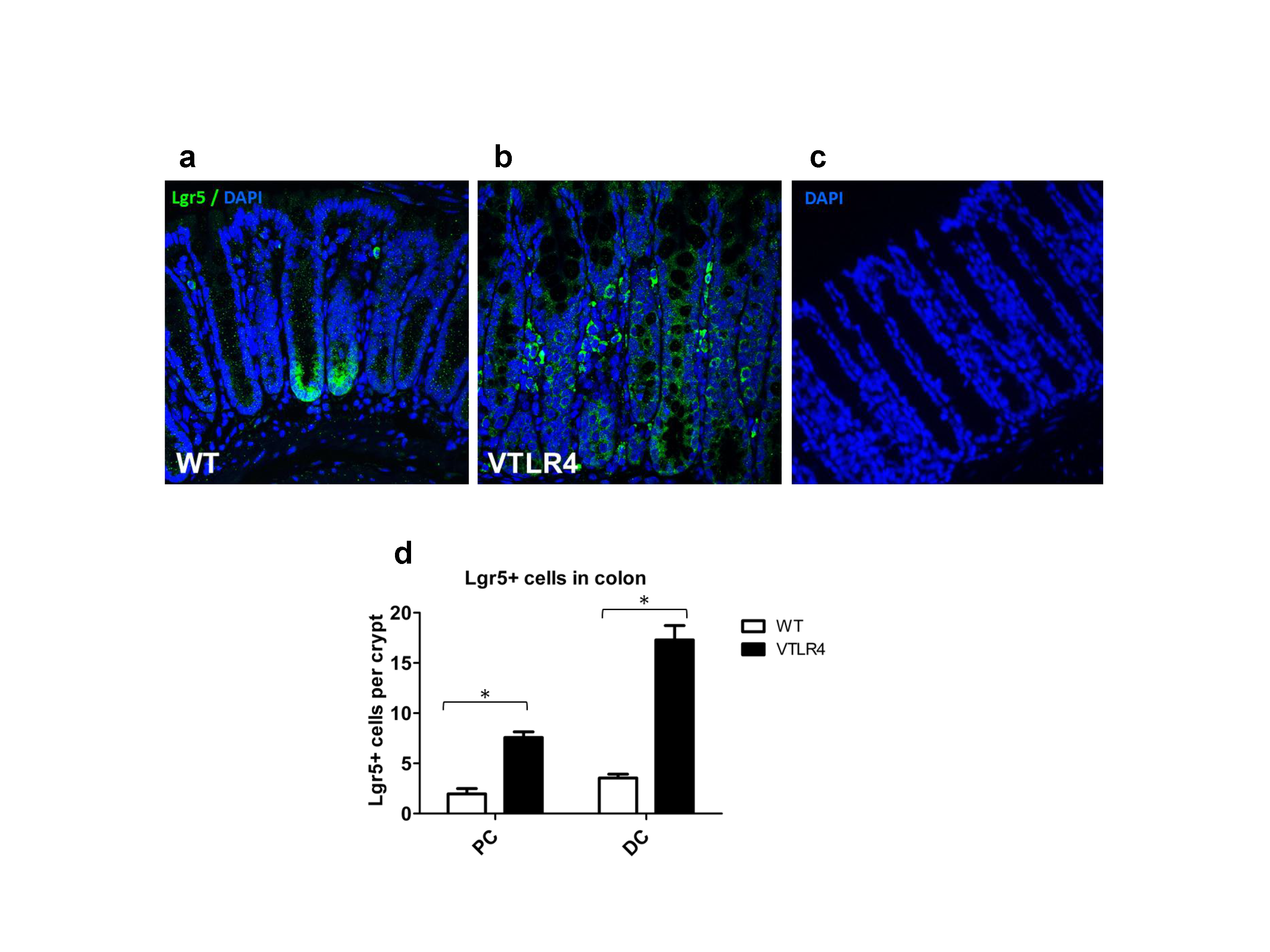

Supplement: Figure S3 — Formalin-fixed paraffin embedded tissues from (a) WT Lgr5-EGFP mice (B6.129P2-Lgr5tm1(cre/ESR1)Cle/J) and (b) villin-TLR4 Lgr5-EGFP mice (villin-TLR4×B6.129P2-Lgr5tm1(cre/ESR1)Cle/J) were cut to detect Lgr5 expression by immunofluorescent staining. We found that in Lgr5+ crypts from the distal colon, Lgr5+ cells were restricted to the bottom of the colonic crypt in WT Lgr5-EGFP mice while Lgr5+ cells in the positive crypts of villin-TLR4 Lgr5-EGFP mice (villin-TLR4×B6.129P2-Lgr5tm1(cre/ESR1)Cle/J) were present throughout the height of the crypt. Lgr5+ cells are shown in green and nuclear counterstaining with DAPI in blue (63x). c) Negative control without primary antibody (anti-EGFP) ruled out background staining from the Lgr5-EGFP staining experiment. d) Graph of Lgr5+ cells in crypts with Lgr5-EGFP expression. Villin-TLR4 mice show a higher amount of Lgr5+ cells than WT mice, in both the proximal (WT: 1.95±0.54 n = 20; VTLR4: 7.57±0.57 n = 7; *p<0.0001) and distal colon (WT: 3.55±0.37 n = 20; VTLR4: 17.30±1.426 n = 20; *p<0.0001). (TIF) [file pone.0063298.s003.tif]

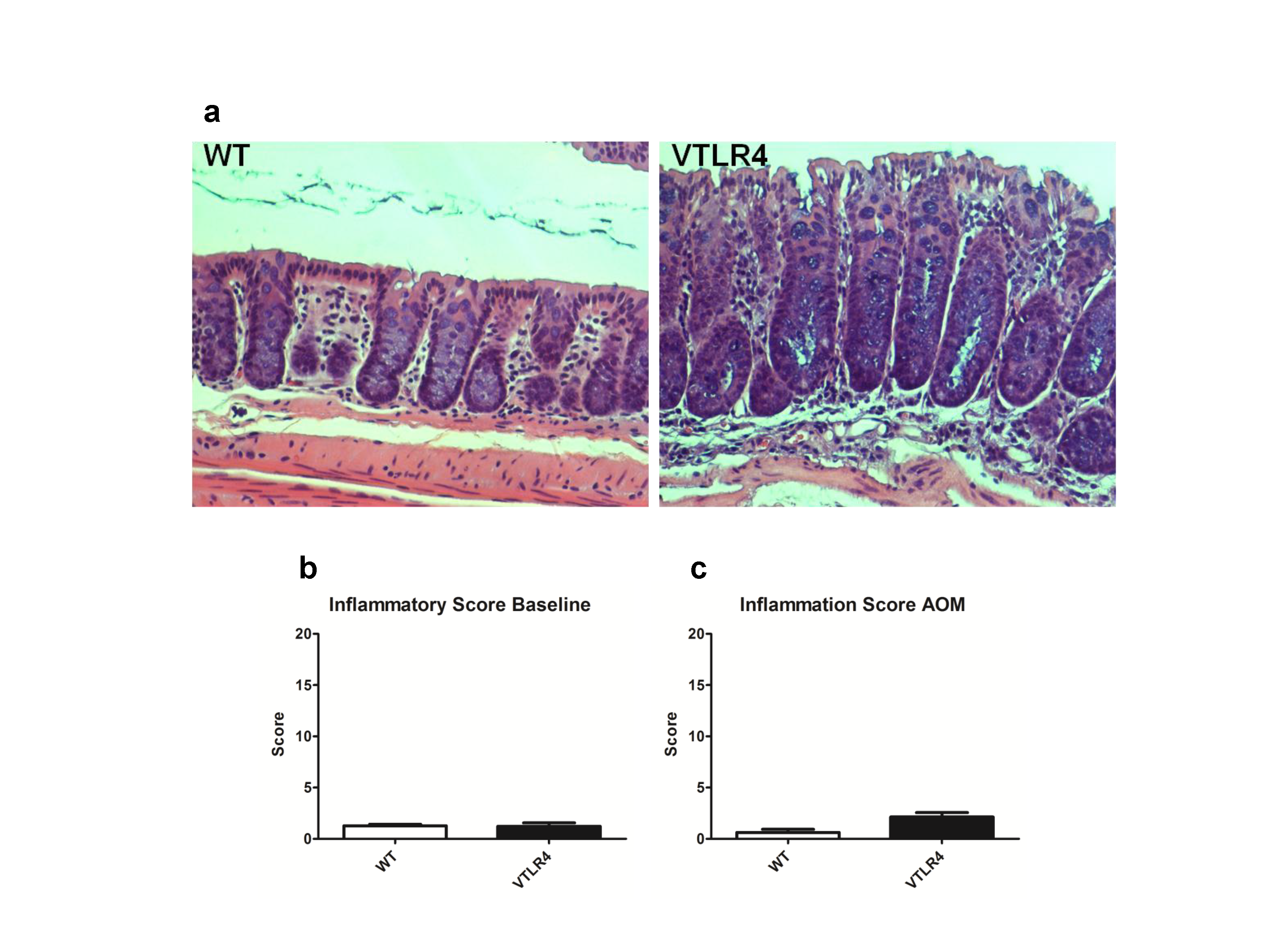

Supplement: Figure S4 — a) Villin-TLR4 (VTLR4) mice do not have baseline inflammation compared to their wild-type (WT) littermates. However VTLR4 show longer colonic crypts compared to WT mice. Both images were taken under the same microscope objective (20x). b) Inflammation was scored in Villin-TLR4 and wild-type mice at baseline and after AOM treatment. There were no significant differences between the two strains neither at baseline (WT: 1.277±0.1468 N = 3, VTLR4: 1.223±0.3359 N = 3, p = 0.320) nor after AOM treatment (WT: 0.6250±0.3146 N = 4, VTLR4: 2.134±0.4334 N = 5, p = 0.503). The inflammatory score can go from 0 to 20, and has into account: crypt damage (0–4), acute inflammation (0–4), edema (0–3), necrosis/ulceration (0–3), chronic inflammation (0–3) and epithelial regeneration (0–3). (TIF) [file pone.0063298.s004.tif]

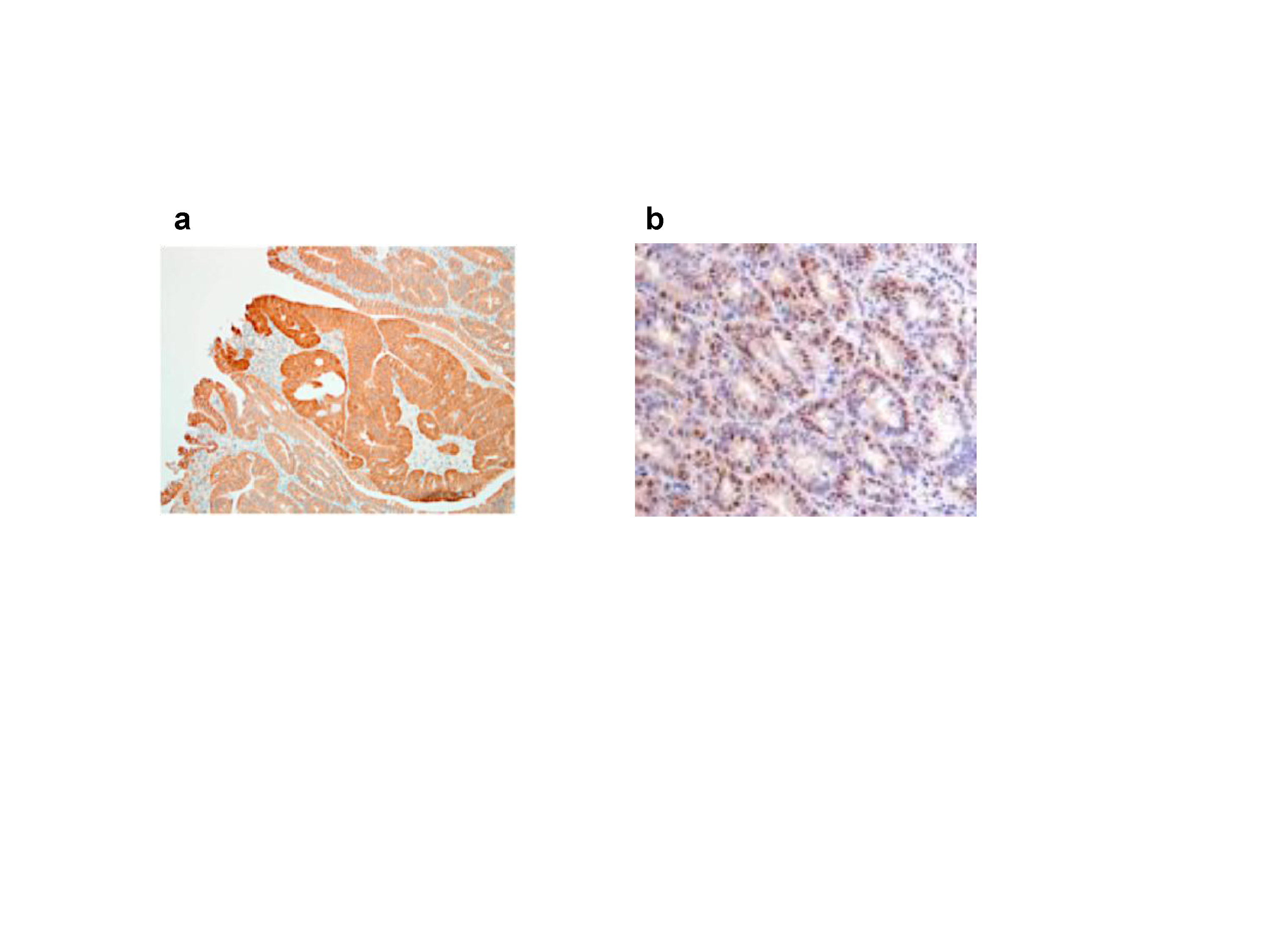

Supplement: Figure S5 — a) Spontaneous duodenal adenomas in villin-TLR4 mice stain for nuclear and cytoplasmic β-catenin (brown), by immunohistochemistry. b) These adenomas are also characterized by cyclin-D1 positive epithelial cells (brown). (TIF) [file pone.0063298.s005.tif]

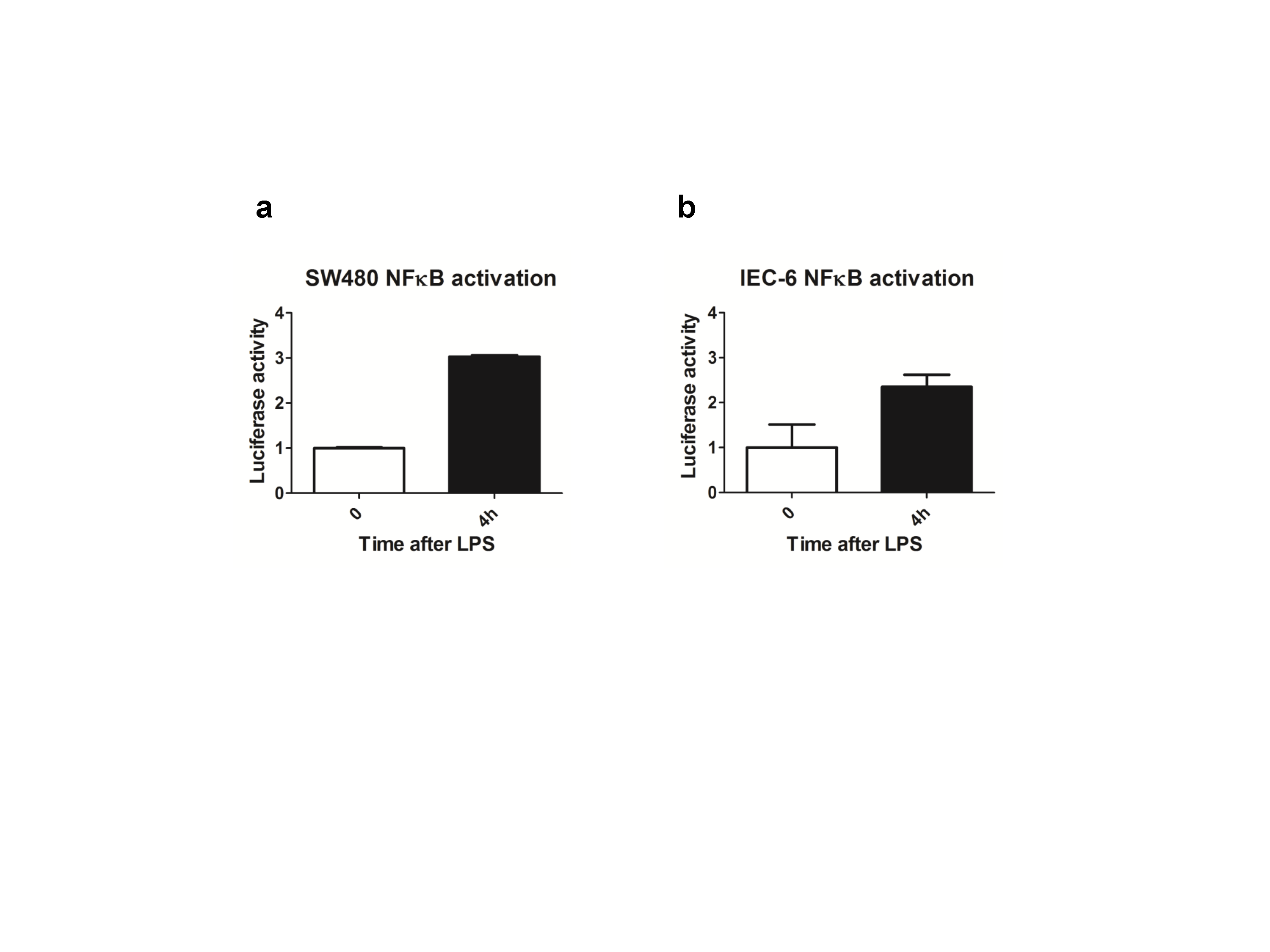

Supplement: Figure S6 — Graphs showing NFκB induction after LPS stimulation in a) SW480 cells and b) IEC-6 cells, measured by NFκB reporter luciferase assay. (TIF) [file pone.0063298.s006.tif]

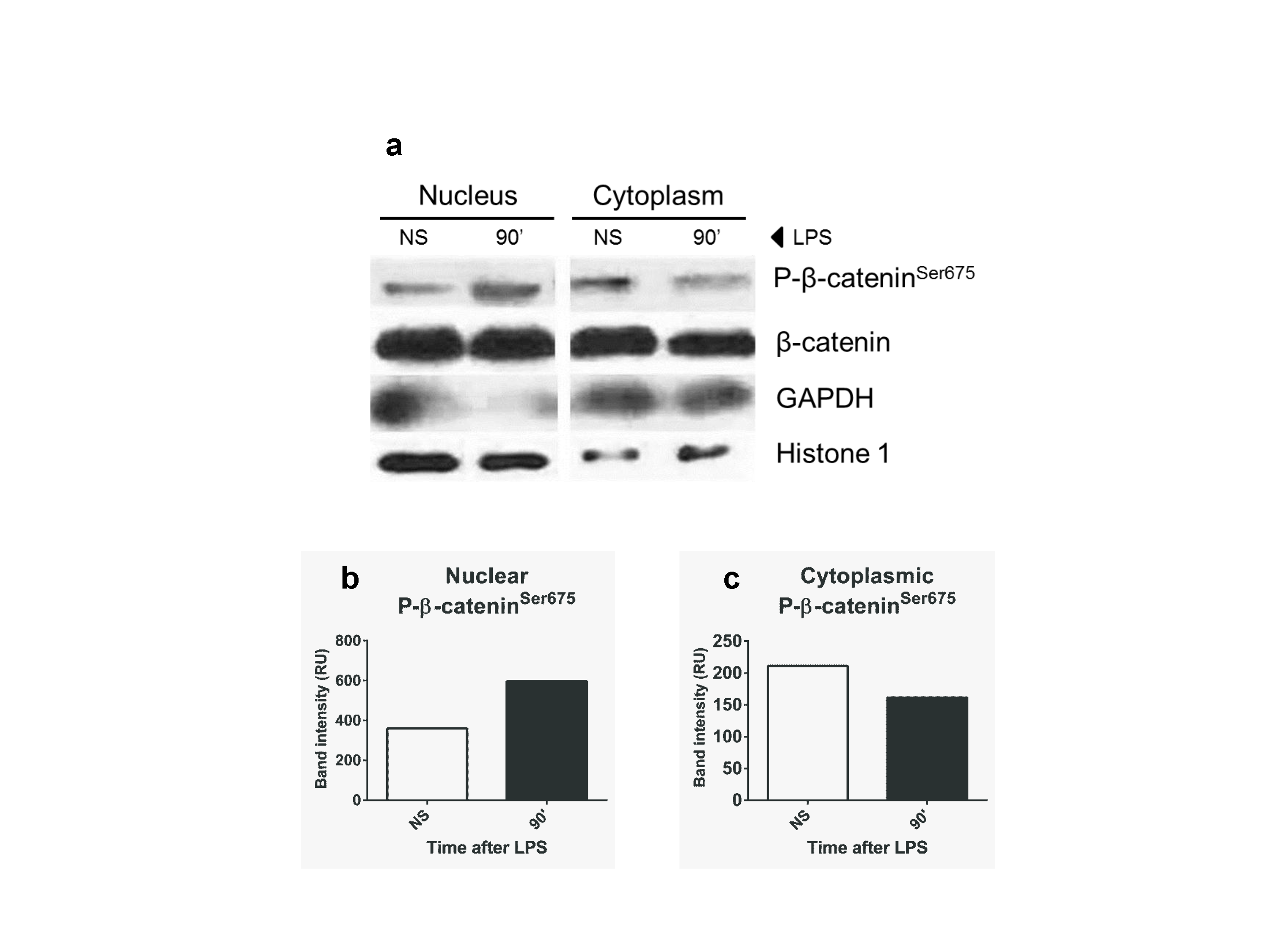

Supplement: Figure S7 — a) Western blot analysis in SW480 nuclear fractions harvested 90 minutes after LPS stimulation. LPS-treated cells show increased phosphorylation of β-catenin at Ser675 (P-β-cateninSer675) in the nuclear fraction. Conversely, the amount of P-β-cateninSer675 decreases in the cytoplasmic fraction, suggesting β-catenin translocation into the nucleus; b) and c) show the densitometry analysis of both cellular fractions. (TIF) [file pone.0063298.s007.tif]

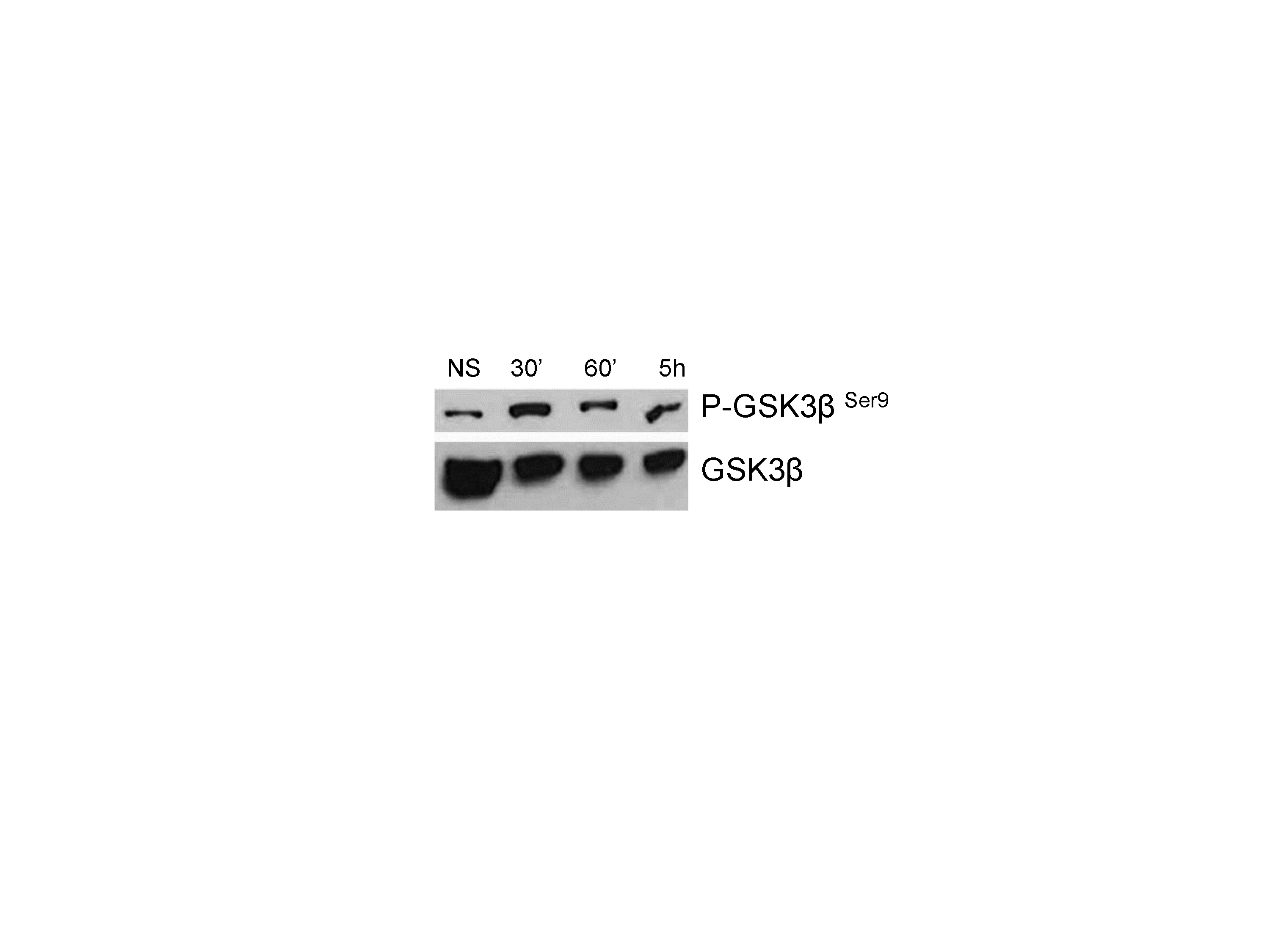

Supplement: Figure S8 — IEC-6 cells respond to LPS with phosphorylation of GSK-3β at Serine 9 (P-GSK3βSer9). Total GSK3β is shown as loading control. (TIF) [file pone.0063298.s008.tif]

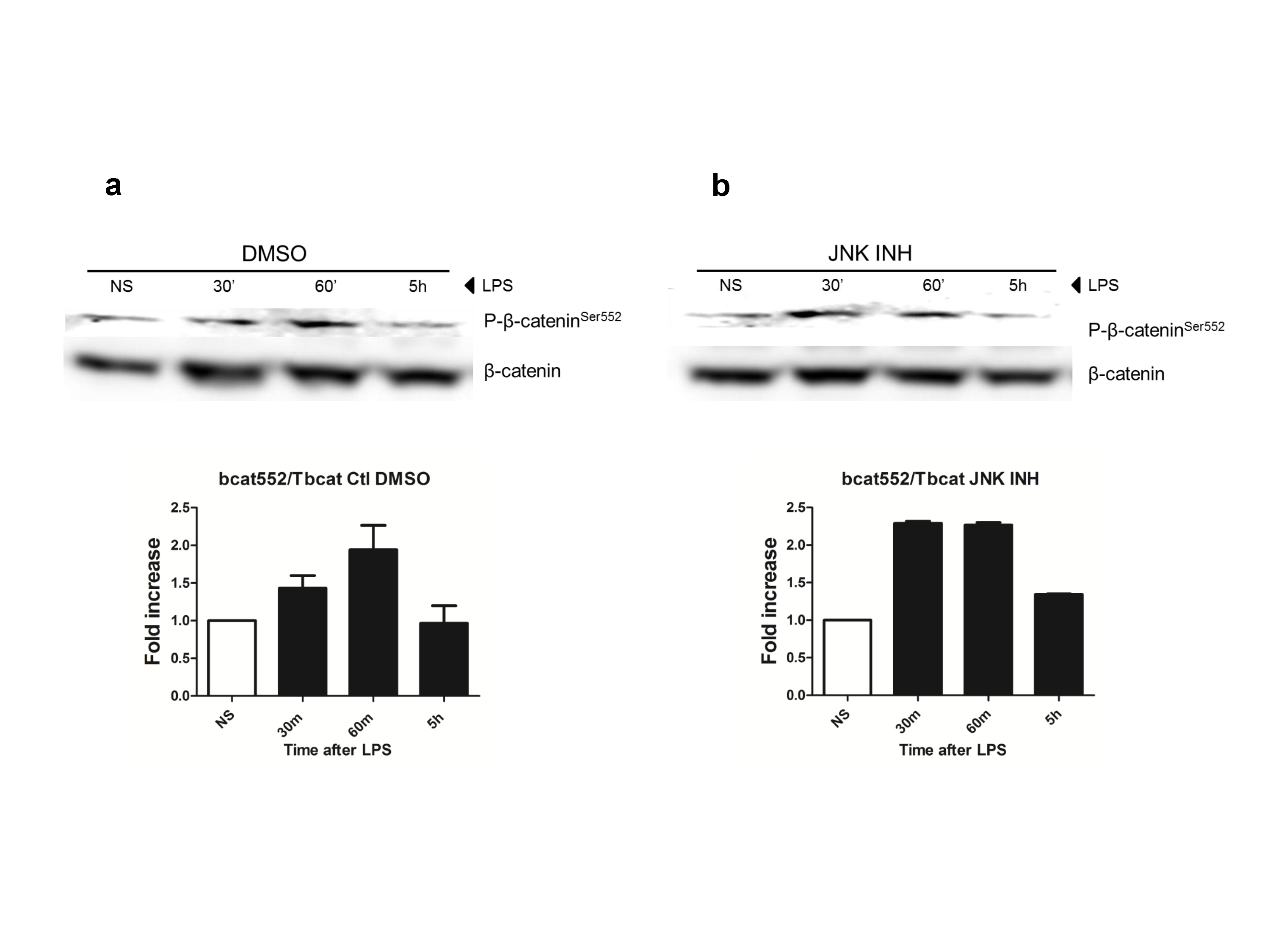

Supplement: Figure S9 — TLR4-mediated β-catenin activation is not affected by JNK signaling in intestinal epithelial cells. IEC-6 cells stimulated with LPS show an increase of β-cateninSer552 phosphorylation in the absence (a) or presence of the SP600125 JNK inhibitor (b). (TIFF) [file pone.0063298.s009.tiff]
